# Icarisid Ⅱ modulates mitochondrial dynamics for anti-HBV activity

**DOI:** 10.3389/fphar.2025.1544714

**Published:** 2025-04-01

**Authors:** Zhengyun Liu, Haiyan Yang, Juan Wen, Dongyan Xiao, Wan Yu, Guo Luo, Qihai Gong, Huan Wang

**Affiliations:** ^1^ Key Laboratory of Infectious Disease and Biosafety, Provincial Department of Education, Zunyi Medical University, Zunyi, Guizhou, China; ^2^ Department of Basic Teaching, Zunyi Medical and Pharmaceutical College, Zunyi, Guizhou, China; ^3^ Key Laboratory of Basic Pharmacology of Ministry of Education and Joint International Research Laboratory of Ethnomedicine of Ministry of Education, Zunyi Medical University, Zunyi, Guizhou, China

**Keywords:** hepatitis B virus, HepG2.2.15 cell, mitochondrial dynamic, ROS, reactive oxygen species, icariside II

## Abstract

**Introduction:**

To investigate the potential anti-hepatitis B virus (HBV) activity of Icariside Ⅱ (ICS Ⅱ), and elucidate its underlying mitochondrial dynamics mechanisms.

**Methods:**

The study employed *in vivo* and *in vitro* assays to evaluate anti-HBV effects of ICS Ⅱ. An HBV replicating mouse model was established through hydrodynamic injection of pAAV/HBV1.2, the impact of ICS Ⅱ on HBV replication and liver toxicity was assessed. *In vitro* cell-based assays used HBV-positive HepG2.2.15 cells. Cytotoxicity was determined with CCK-8 assay, while ELISA and qPCR were employed to measure HBsAg, HBeAg, and HBV DNA levels. The livers of ICS II-treated HBV-infected mice were taken for transcriptome sequencing to screen for different genes, and the results were verified by Western Blot. Mitochondrial morphology and dynamics were visualized using confocal imaging and transmission electron microscopy. Key protein expressions related to mitochondrial fission and fusion were analyzed via WB. Intracellular ROS generation was assessed using fluorescence staining.

**Results:**

The study found that ICS Ⅱ exhibited significant anti-HBV effects both *in vivo* and *in vitro*. The results of RNA-Seq indicated that ICS Ⅱ modulated the mRNA levels of Fisl, a protein associated with mitochondrial dynamics, during the anti-HBV response. It induced mitochondrial fragmentation and enhanced mitochondrial motility in HBV-positive cells. Notably, key proteins associated with mitochondrial fission and fusion demonstrated alterations favoring fission. Furthermore, ICS Ⅱ effectively reduced ROS production in HBV-positive cells.

**Conclusion:**

ICS Ⅱ exhibits significant anti-HBV potential through its regulation of mitochondrial dynamics and ROS production.

## 1 Introduction

Hepatitis B Virus (HBV) infection poses a grave risk, potentially causing chronic hepatitis, liver fibrosis, cirrhosis, and hepatocellular carcinoma, significantly endangering patient lives. While Interferon and nucleotide analogues serve as primary therapeutic agents, delivering robust antiviral effects, their use is marred by substantial adverse reactions. Interferon treatment exhibits a low response rate alongside significant adverse effects. The extended application of nucleoside analogues raises concerns of drug resistance and toxic side effects. Curtailing hepatitis B progression and complications necessitates novel strategies to disrupt HBV infection and replication. Maximizing the inhibition of HBV replication is also important in achieving this goal.

Mitochondria, pivotal organelles ubiquitously present in cells, function as “powerhouses,” generating cellular energy. Their constant division and fusion processes yield various forms, such as tubular networks or punctate structures, collectively known as mitochondrial dynamics. These dynamics profoundly influence mitochondrial count, size, arrangement, and energy metabolism within cells (Wang et al., 2020). Extensive research underscores the pivotal role of mitochondrial dynamics in diverse diseases, nervous system disorders, and bacterial or viral infections. Dysregulations in these dynamics can trigger mitochondrial dysfunction, profoundly impacting host cellular function (Chan, 2020; Khan et al., 2020). Remarkably, viruses demonstrate distinct responses upon perturbing mitochondrial dynamics during different infection phases. ZIKV (PRVABC59) infection of JEG-3 trophoblast cells manipulates mitochondrial dynamics, mitophagy, and formation of mitochondria-derived vesicles (MDVs) (Lee and Shin, 2023). Notably, HBx of genotypes A and G caused strong disruption of mitochondrial morphology (Schollmeier et al., 2024). This alteration is facilitated by the viral HBx protein, underscoring its central role in this process.

*Epimedium*, a traditional Chinese medicinal herb sourced from the *Epimedium* genus, boasts diverse pharmacological effects. Several studies validate the anti-HBV potential of *Epimedium* extract or polypills (Tian et al., 2019; Zheng et al., 2023). A prominent constituent within *Epimedium*, Icariside Ⅱ (ICS Ⅱ), showcases broad activities, encompassing anti-oxidative and anti-inflammatory properties (Zheng et al., 2020). This study unveils the *in vitro* and *in vivo* anti-HBV efficacy of ICS Ⅱ, while dissecting its influence on mitochondrial dynamics governing HBV replication. The insights advocate for ICS Ⅱ as a promising supplementary approach to control HBV infections and associated diseases.

## 2 Materials and methods

### 2.1 Animals

Male C57BL/6 mice (6–8 weeks old) were procured from SPF (Beijing) Biotechnology Co., Ltd. (Beijing, China; Certificate No. SCXK (jing) 2016-0002). Ethical considerations were upheld in all animal experiments, as approved by the Experimental Animal Ethics Committee of Zunyi Medical University (Guizhou, China). The research adhered to the international guidelines for the care and utilization of laboratory animals, as stipulated by the US National Institutes of Health.

### 2.2 Cell culture

HBV-positive HepG2.2.15 cells were cultured in DMEM high glucose medium (Livning) supplemented with 10% fetal bovine serum (FBS) (Livning), 0.4 mg/mL G418, 100 mg/mL streptomycin and 100 IU/mL penicillin at 37°C, 5% CO_2_ in a humidified incubator.

### 2.3 Reagents

ICS Ⅱ (purity≥98%) was purchased from Nanjing Xinhou Biotechnology Co., Ltd. Entecavir (ENT) dispersible tablets were obtained from Jiangxi Qingfeng Pharmaceutical Co., Ltd.

### 2.4 Cell viability assay

Cell viability was analyzed by the Cell Counting Kit-8 (CCK-8) Assay. Briefly, HepG2.2.15 cells were seeded in a 96-well plate (1 × 10^4^ cells per well) for 24 h and cultured with fresh medium containing various concentrations of ICS Ⅱ or entecavir. Following 72 h of incubation, 10 μL CCK-8 reagent was added and the plates were incubated at 37°C for 2 h. Subsequently, the value of optical density (OD) of the cells was measured at 450 nm using a Multiskan Spectrum (Thermo). The impact of the drugs on cell viability was assessed as the percentages of viability compared with the control cells which were assigned as having 100% viability.

### 2.5 Cell-based anti-HBV activity assay

HepG2.2.15 cells were seeded in 6-well plates (5 × 10^5^ cells per well) for 24 h. Subsequently, the cells were treated with various concentrations of ICS Ⅱ or entecavir for 3 days. Cell culture supernatants were collected for measuring HBsAg and HBeAg by using a commercial ELISA kit (Zhongshan Bio-Engineering) according to the manufacturer’s protocol. Furthermore, HBV DNA levels were quantified using a commercial HBV nucleic acid test kit (PCR fluorescence probe method) (Daan Gene) according to the manufacturer’s instructions.

### 2.6 *In vivo* anti-HBV activity assay

C57BL/6 mice were treated with hydrodynamic injection of pAAV/HBV1.2 (adeno-associated virus carrying 1.2 copies of the HBV genome), following previously described procedures (Bian et al., 2017; Liu et al., 2023). Founder mice were selected based on serum HBV DNA analysis at 3 days post-injection, with preference for those producing HBV DNA levels exceeding 10^3^ copies/mL. In subsequent experiments, the mice were divided into four groups (n = 6/group): control mice (without pAAV-HBV1.2) receiving sterile saline (0.9%), vehicle mice receiving sterile saline (0.9%), and other mice treated with ENT (0.5 mg/kg) or ICS Ⅱ (20 mg/kg) (intragastric administration, once daily) for 20 days.

Mice were euthanized at 23 days, and blood samples were collected for analysis. Commercial enzyme-linked immunosorbent assay (ELISA) kits (Zhongshan Bio-Engineering) were used for detecting HBsAg and HBeAg, following the manufacturer’s protocol. Quantification of HBV DNA levels was executed through a commercial HBV nucleic acid test kit (PCR fluorescence probe method) (Daan Gene), in accordance with the manufacturer’s instructions. Concurrently, mouse livers were preserved in 10% formalin, followed by standard hematoxylin and eosin (HE) staining procedures^[100]^.

### 2.7 RNA sequencing screening of differentially expressed genes (DEGs)

Livers samples collected at 23 days (vehicle and ICS Ⅱgroup). The RNA was extracted using Trizol, and the RNA concentration and purity were confirmed After library preparation, sequencing of samples was performed using Hiseq PE150. Reads were filtered by quality control with FastQC, then clean data were obtained by trimming adapters, quality control alignment was done with qualimap. TMM was used to standardize the read count data, followed by DEG.seq.

### 2.8 Confocal imaging of mitochondrial morphology and image analysis

Live cell imaging using LSM900 confocal microscopy (ZEISS) was conducted for fluorescent dye loading. Cells were incubated with 500 nM MitoTracker Deep Red FM (Invitrogen) for 30 min at 37°C for visualization of mitochondrial morphology and motility. MitoTracker Deep Red FM was excited at 644 nm and the emitted fluorescence was collected at 665 nm.

### 2.9 Detection of mitochondrial morphology by transmission electron microscopy (TEM)

Cells pellets were harvested and fixed for 2 h using a 2.5% glutaraldehyde solution in phosphate buffer. Following fixation, the cells underwent phosphate buffer rinsing and were subsequently post-fixed for 1 h using 1% osmic acid. Sequential ethanol solutions of increasing concentrations (30%–100%) were used for cell dehydration for 5 min at room temperature. Subsequently, the cells were embedded in epon812 resin, sectioned using a UC-7 ultramicrotome (Leica), and subjected to 2% uranyl acetate staining for 30 min. Observations and images were acquired using a transmission electron microscope (Japan).

### 2.10 Western blot analysis

The protein was extracted from cultured cells using a protein lysis buffer. Protein samples were resolved using SDS-polyacrylamide gel electrophoresis (SDS-PAGE; 150 V, 90 min). Electro-transferring of protein bands onto nitrocellulose membranes (350 mA, 120 m) ensued. Subsequently, membranes were blocked with TBS contained 0.1% Tween 20% and 5% milk for 1 h. The primary antibodies used are β-actin (Solarbio K200058M; dilution: 1/5000), Drp1(CST #8570; dilution: 1/1000), p-Drp1 (ser 616, CST #3455; dilution: 1/1000), p-Drp1 (ser 637, Abcam ab193216; dilution: 1/750), Opa1(CST #80471; dilution: 1/1000), Fis1(Invitrogen PA5-106271; dilution: 1/1000), Mfn1(Santa Cruz #50330; dilution: 1/500) and Mfn2(CST, #11925S; dilution: 1/1000). The primary antibodies were diluted in 5% BSA-TBST-0.05% NaN_3_. After overnight incubation with the primary antibody at 4°C, membranes were washed thrice in TBST buffer and then incubated with horseradish peroxidase-conjugated secondary antibodies at room temperature for 2 h. Results were visualized with ECL reagents (Solarbio). Densitometry evaluation was conducted using ImageJ software.

### 2.11 ROS level evaluation

The fluorescent dyes ROS Brite™ 570 (AAT) was used to evaluate total cellular ROS production level in cells. For detecting total cellular ROS production level, cells were incubated with 5 μM ROS Brite™ 570 in medium for 30 min at 37°C. Fluorescence intensity of ROS Brite™ 570 was evaluated using a ZEISS LSM900 confocal microscope. ROS Brite™ 570 was excited at 556 nm with the fluorescent images collected at 566 nm.

### 2.12 Statistical analysis

The results were presented as mean value ±standard deviation (SD). All the data were analyzed using the SPSS 29.0, using independent sample T-test and a one-way analysis of variance (ANOVE), followed by LSD or Dunnett’s T3. *P* value <0.05 was considered to indicate a statistically significant difference.

## 3 Results

### 3.1 Anti-HBV effects of ICS Ⅱ *in vitro*

**FIGURE 1:**
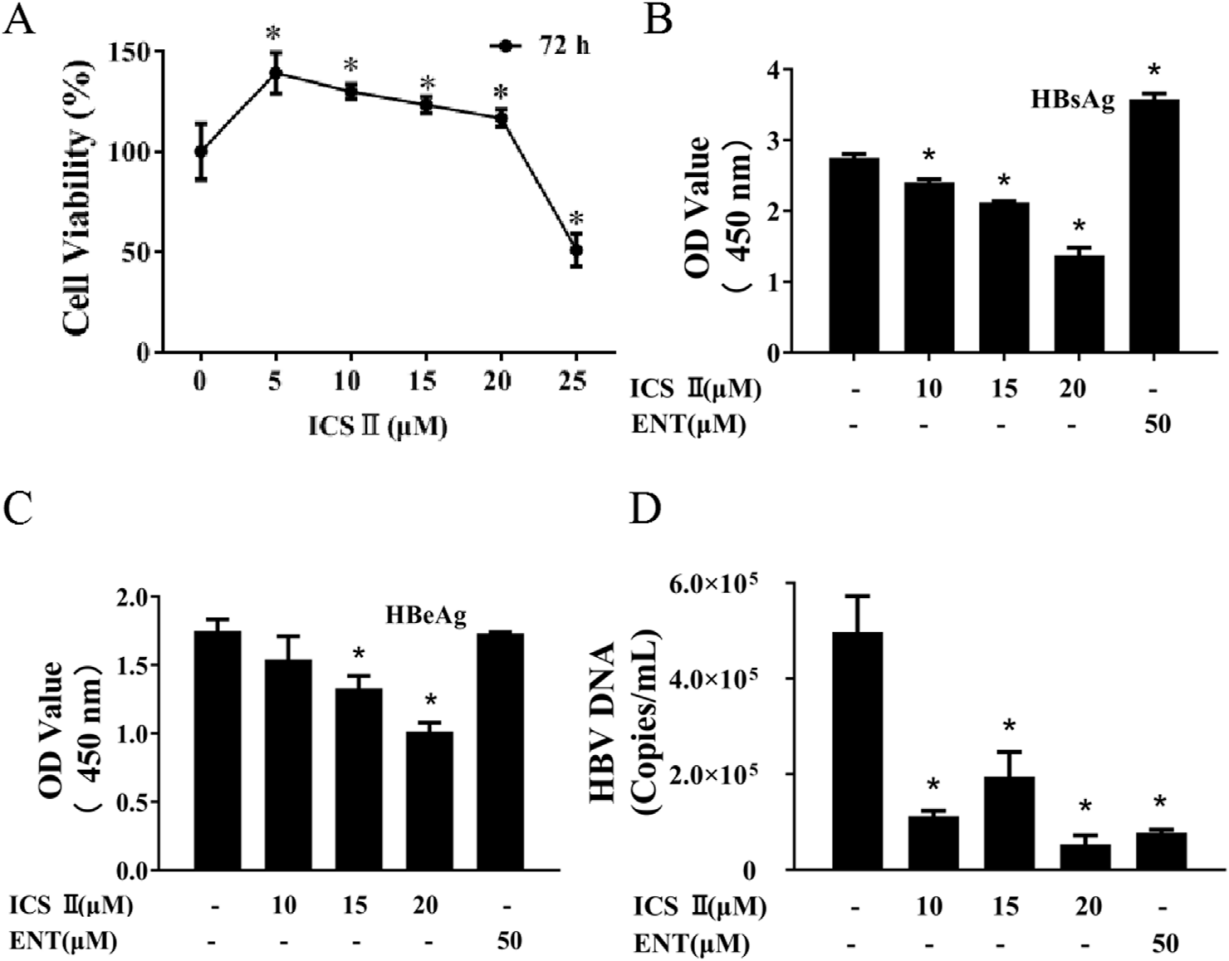
ICS Ⅱ demonstrates anti-HBV activities *in vitro*. HepG2.2.15 cells were exposed to varying concentrations of ICS II for a duration of 72 h. The viability of these cells post-treatment was evaluated using the CCK8 assay **(A)**. Subsequently, cell culture supernatants were harvested to determine the levels of HBsAg **(B)**, HBeAg **(C)** in HepG2.2.15 cells using an ELISA kit. Additionally, the presence of HBV DNA **(D)** in HepG2.2.15 cells was ascertained using an HBV nucleic acid test kit after the 72-hour ICS II treatment. The presented results are the mean values derived from three biological replicates, with error bars indicating standard deviations. *P < 0.05 signifies a notable difference from the control group.

To explore whether ICS Ⅱ exhibited antiviral activities against HBV, HepG2.2.15 cells were used *in vitro*. At first, the CCK-8 assay was performed to assess the cytotoxicity of ICS Ⅱ. Cells were treated with varying concentrations of ICS Ⅱ 0, 5, 10, 15, 20, 25 μM for 72 h, and ICS Ⅱ 25 μM decreased cell viability (Figure 1A). In the assessment of anti-HBV activity, ICS Ⅱ displayed significant inhibition of HBsAg, HBeAg, and HBV DNA. ICS Ⅱ's inhibitory effect on HBV in HepG2.2.15 was even more pronounced than that of ENT (Figures 1B–D). Collectively, these findings underscore the *in vitro* anti-HBV potential of ICS Ⅱ.

### 3.2 ICS Ⅱ exhibited anti-HBV activity in C57BL/6 mice

**FIGURE 2:**
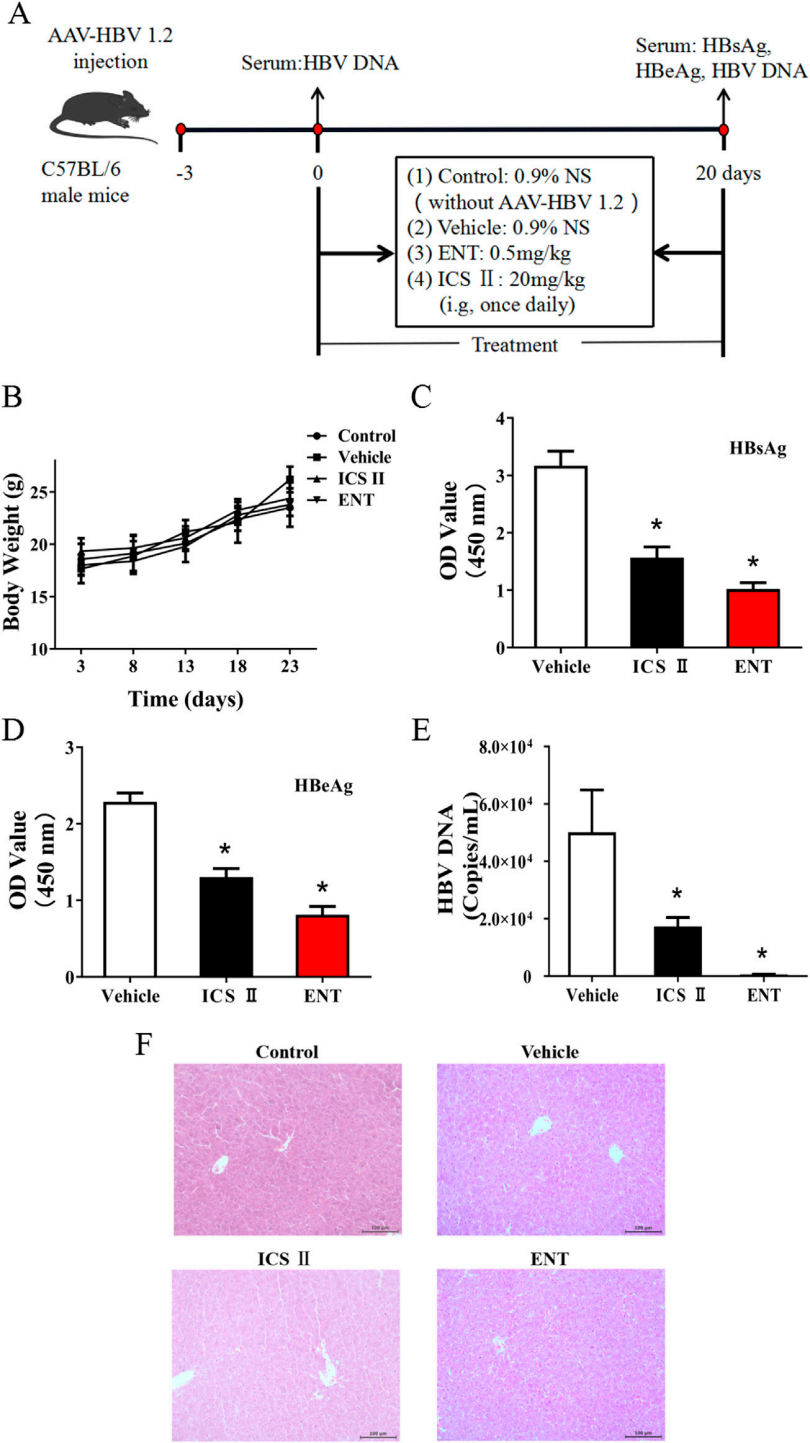
Anti-HBV effects of ICS Ⅱ in C57BL/6 mice. This figure provides an overview of the *in vivo* assay described in the manuscript. C57BL/6 mice were inoculated with pAAV/HBV1.2 and subsequently treated with either sterile saline (0.9%), ICS Ⅱ(20 mg/kg), or ENT (0.5 mg/kg) **(A)**. The body weight of the mice was monitored **(B)**, and after 20 days of treatment, the levels of HBsAg **(C)**, HBeAg **(D)**, HBV DNA **(E)**, and liver HE **(F)** stain were assessed. *P < 0.05 denotes a significant deviation from the control group. The significance was ascertained using a one-way ANOVA, complemented by Dunnett’s T3 multiple comparison test with a single pooled variance.

The effect of ICS Ⅱ on HBV replication was assessed in a mouse model with HBV replication, established through hydrodynamic injection (Figure 2A). ENT was used as a positive control. Throughout the experiment, no significant differences in mice weight were observed among the groups (Figure 2B). Following 20 days of treatment, both ICS Ⅱ and ENT significantly lowered serum HBsAg, HBeAg, and HBV DNA levels compared to the vehicle group (*P* < 0.05) (Figures 2C–E), and ICS Ⅱ did not exhibited significant liver toxicity. The data indicated that ICS Ⅱ inhibited HBV DNA synthesis *in vivo*, although its efficacy was slightly lower than ENT.

### 3.3 RNA-seq showed Fis1 was involved in the process of ICS II inhibited HBV replication

ICS II has a significant inhibitory effect on HBV replication *in vitro* and *in vivo*, but its mechanism is still unclear. HBV infection causes substantial harm to mitochondrial activity, and HBV can disrupts mitochondrial dynamics (Lin et al., 2024; Mansouri et al., 2018), and mitochondrial DNA is reportedly targeted by HBV integration (Giosa et al., 2023). In RNA-Seq, the results show significant enrichment in pathways related to: (1) mitochondrial function: including cellular metabolic process and ubiquitin mediated proteolysis, which are closely linked to mitochondrial dynamics. (2) viral replication: such as the PI3K-Akt signaling pathway, which has been implicated in HBV replication (Lin et al., 2024) (Supplementary Figure S1). These findings support the hypothesis that ICS II’s modulation of mitochondrial dynamics and host cell metabolism contributes to its anti-HBV effects. There are 25 DEGs related to mitochondria with a 1.5 fold change, of which the Fis1 was the most closely related to mitochondria (Table 1). It mainly participates in mitochondrial division and as a key protein in mitochondrial dynamics.

**TABLE 1:** DEGs related to mitochondria.

GeneID	Gene Name	Mean TPM (vehicle)	Mean TPM (ICSII)	log2 Fold Change	Result
ENSMUSG00000022956	Atp5o	37.4	0.0001	18.51	up
ENSMUSG00000034566	Atp5h	12.52	0.0001	16.93	up
ENSMUSG00000027282	Mtch2	5.82	0.0001	15.83	up
ENSMUSG00000079659	Tmem243	5.38	0.05	6.75	up
ENSMUSG00000025781	Atp5c1	26.98	0.93	4.86	up
ENSMUSG00000025428	Atp5a1	64.68	5.11	3.66	up
ENSMUSG00000026621	Marc1	75.5	5.99	3.66	up
ENSMUSG00000029486	Mrpl1	10.21	1.02	3.32	up
ENSMUSG00000015112	Slc25a13	14.76	3.85	1.94	up
ENSMUSG00000061904	Slc25a3	38.45	10.2	1.91	up
ENSMUSG00000026621	Marc1	100.47	28.39	1.82	up
ENSMUSG00000052337	Immt	6.27	1.88	1.74	up
ENSMUSG00000023723	Mrps23	6.86	2.28	1.59	up
ENSMUSG00000028107	Tars2	5.34	1.8	1.57	up
ENSMUSG00000022890	Atp5j	5.91	17.92	-1.60	down
**ENSMUSG00000019054**	**Fis1**	**4.72**	**15.8**	**-1.74**	**down**
ENSMUSG00000034729	Mrps10	2.74	9.88	-1.85	down
ENSMUSG00000027282	Mtch2	3.86	39.25	-3.35	down
ENSMUSG00000023861	Mpc1	1.63	22.69	-3.80	down
ENSMUSG00000061474	Mrps36	0.19	5.81	-4.93	down
ENSMUSG00000030541	Idh2	0.19	18.66	-6.62	down
ENSMUSG00000026621	Marc1	0.34	126.65	-8.54	down
ENSMUSG00000019082	Slc25a22	0.02	24.7	-10.27	down
ENSMUSG00000029433	Diablo	0.0001	7.49	-16.19	down
ENSMUSG00000027601	Mtfr1	0.0001	45.62	-18.80	down

Bold values represent the genes most closely related to mitochondrial dynamics.

### 3.4 ICS Ⅱ promoted mitochondrial fragmentation and dynamics in HBV-positive cells

**FIGURE 3:**
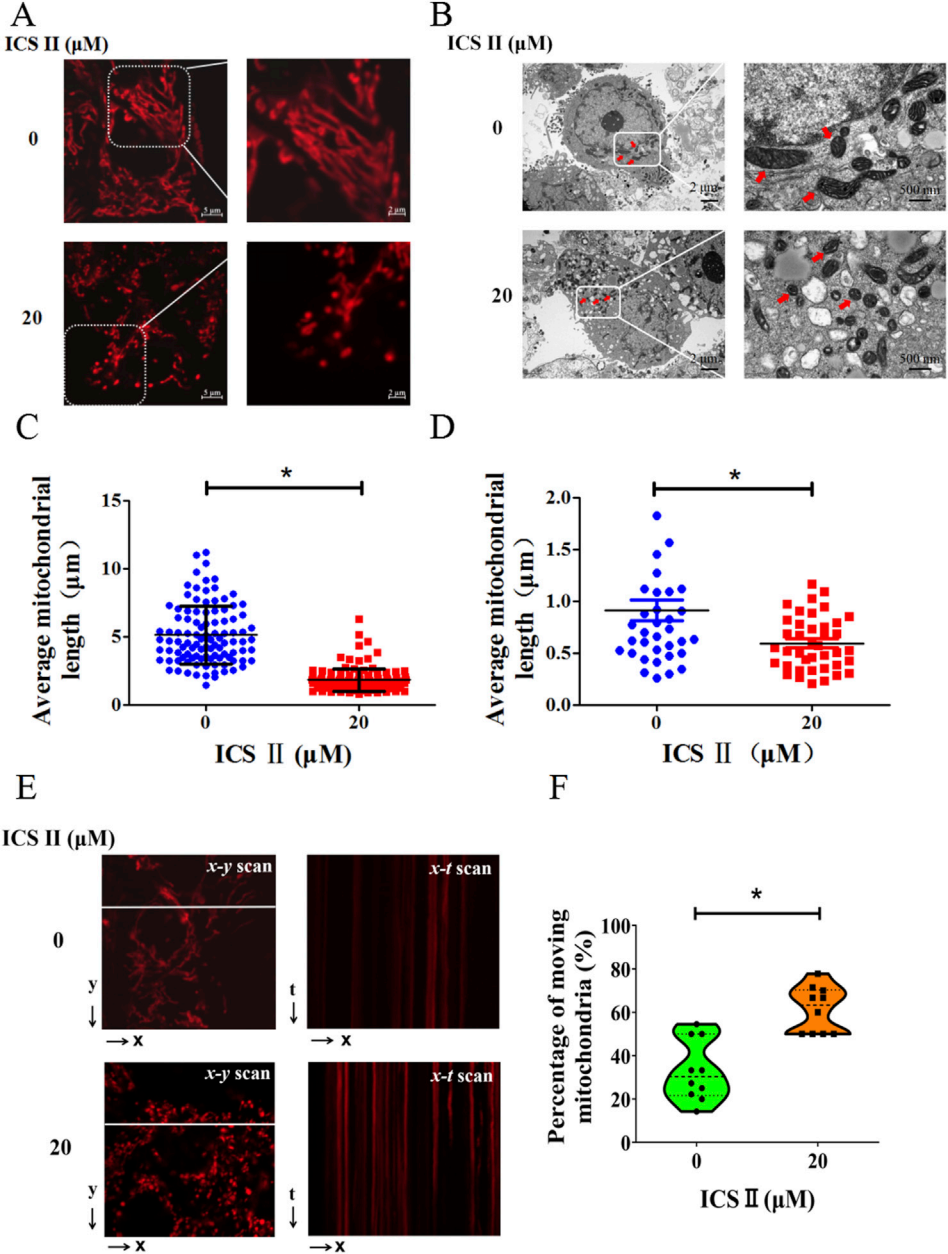
ICS Ⅱ promoted mitochondrial fragmentation and dynamics in HBV-positive cells. For visualizing mitochondria, HepG2.2.15 cells were stained with MitoTracker Deep Red **(A)**. The mitochondrial ultrastructure in these cells was further examined using transmission electron microscopy **(B)**. Notably, the red arrows point to the mitochondria. The graphs in **(C, D)** provide a statistical representation of mitochondrial lengths corresponding to images **(A, B)**. Subsequently, x-t line scan imaging was employed to track the motility of individual mitochondria along a designated scan line, as highlighted by the white dashed lines **(E)**. The motility of these mitochondria was quantified by determining the percentage of mitochondria within a single cell that exhibited movement during the observation period. Graphs **(F)** provide a statistical representation of the percentage of moving mitochondria, corresponding to images **(E)**. The data presented are mean values derived from three biological replicates, with error bars indicating standard deviations. *P < 0.05 signifies a notable difference from the control group.

To comprehend mitochondrial morphology changes during ICS Ⅱ's anti-HBV activity, live cell imaging with MitoTracker Deep Red staining was performed. ICS Ⅱ-treated cells exhibited more fragmented mitochondrial structures compared to the intact mitochondrial networks in the 0 μM group (Figure 3A). The mean mitochondrial length exhibited significantly decreased, a finding supported by observations through transmission electron microscopy (Figure 3B). These outcomes indicate that ICS Ⅱ induces mitochondrial fragmentation in HBV-positive cells.

Subsequently, we investigated the impact of ICS Ⅱ on mitochondrial movement within HBV-positive cells using MitoTracker Deep Red staining. For a quantitative assessment of mitochondrial dynamics, we employed the x-t mode line scan approach. As illustrated in representative images within Figure 3E, the x-y scan mode allowed the collection of images to discern individual mitochondria within a subcellular region. Subsequently, the line scan x-t mode imaging was conducted to monitor the mobility of individual mitochondria in live cells. The degree of mitochondrial mobility was quantified by calculating the percentage of mitochondria within a single cell that exhibited movement during the 75 s recording interval (Figure 3F). Evidently, in comparison to the 0 μM group, the ICS Ⅱ group demonstrated a significant increase in the number of moving mitochondria. These findings provide compelling evidence that ICS Ⅱ effectively accelerates mitochondrial motility in HBV-positive cells.

### 3.5 ICS Ⅱ modulated the expression of key proteins involved in mitochondrial fission and fusion in HBV-positive cells

**FIGURE 4:**
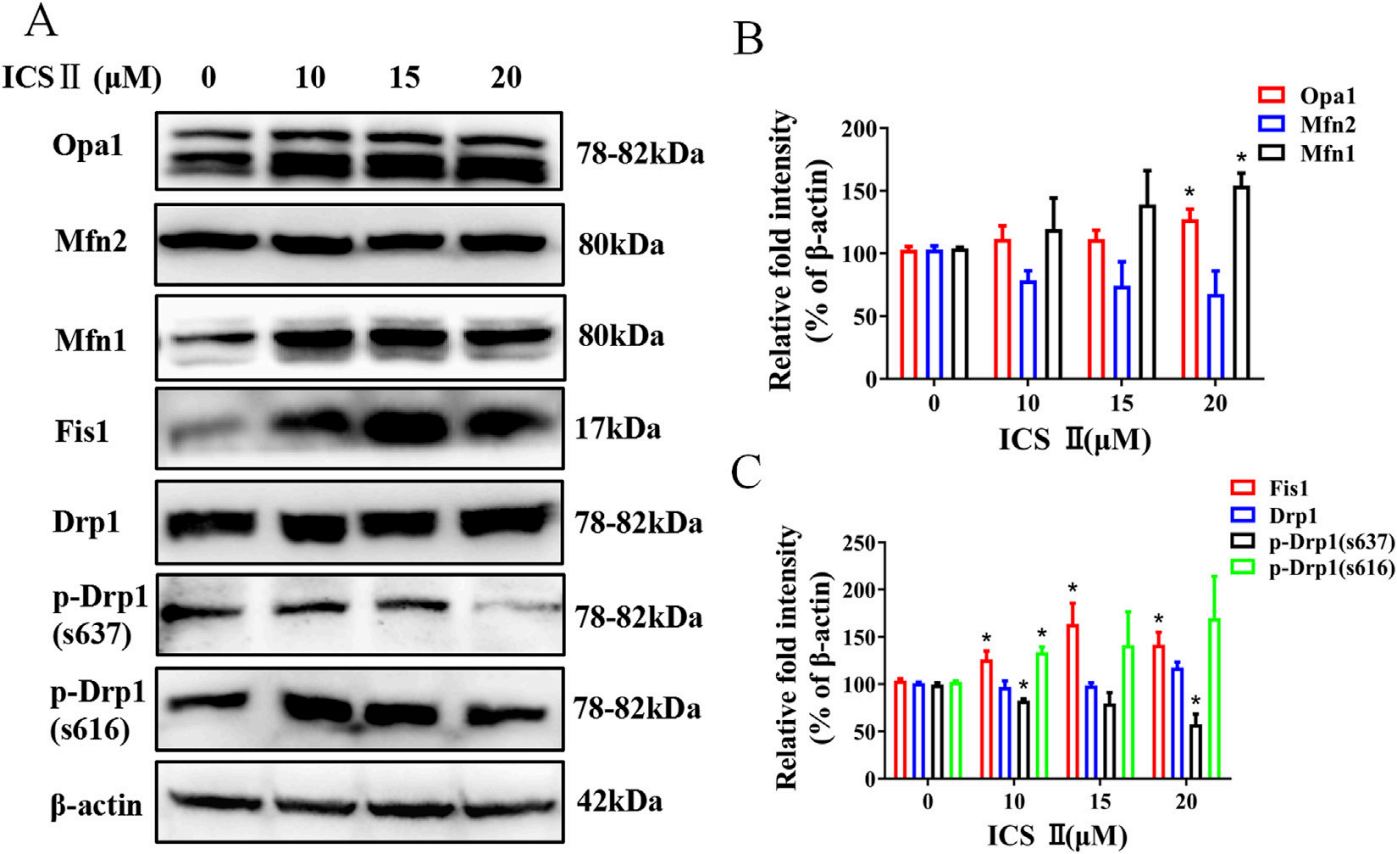
ICS Ⅱ altered the expression levels of key proteins involved in mitochondrial fission and fusion in HBV-positive cells. Western blotting was employed to monitor alterations in proteins associated with mitochondrial fission and fusion, β-actin served as a loading control (**A**). The relative protein intensities were quantified using ImageJ software as shown in panels **(B, C)**. The presented data are mean values derived from three biological replicates, with error bars indicating standard deviations. *P < 0.05 signifies a notable difference from the control group.

Critical to mitochondrial dynamics are key proteins like phosphorylated Dynamin-related protein 1 (p-Drp1) S616 and Fis1, which promote mitochondrial fission, and Optic Atrophy 1 (Opa1), Mitofusin 1 (Mfn1), Mitofusin 2 (Mfn2), and p-Drp1 S637 pivotal for promoting mitochondrial fusion. To ascertain whether ICS Ⅱ directly influences the key proteins associated with mitochondrial dynamics in HBV-positive cells, we conducted immunoblotting analysis to determine the expression levels of these crucial proteins. Although Mfn2 did not display significant changes in the ICS Ⅱ-treated groups, the protein expression levels of Opa1, Mfn1, Fis1 and p-Drp1 S616 were markedly increased, and the protein expression levels of p-Drp1 S637 were markedly decreased (Figure 4).

### 3.6 ICS Ⅱ reduced the ROS production in HBV-positive cells

**FIGURE 5:**
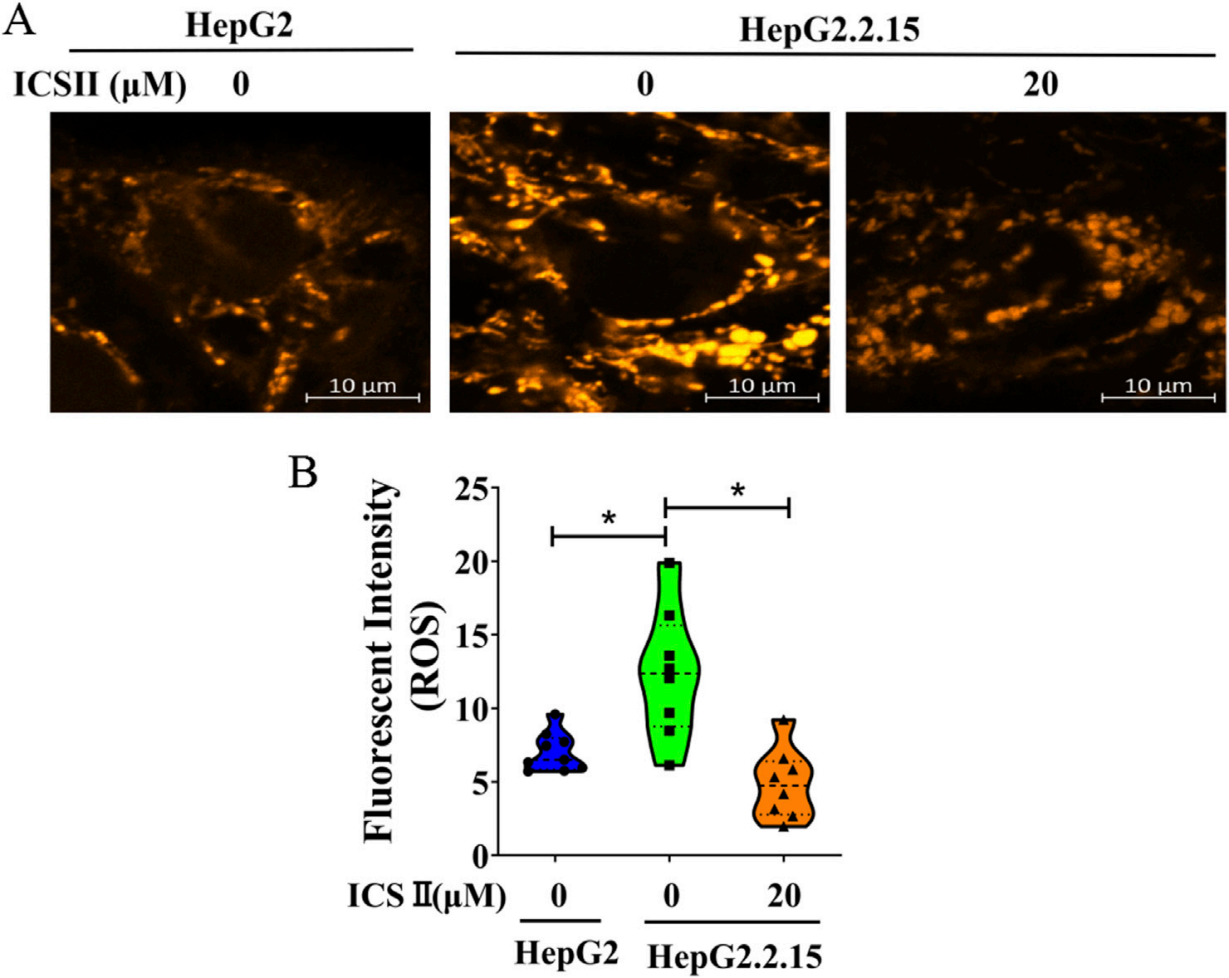
ICS Ⅱ diminishes ROS production in HBV-positive cells. Intracellular ROS levels in HepG2.2.15 cells were assessed using the ROS Brite™ 570 staining method. Graphs **(B)** provide a statistical representation of the average fluorescence intensity corresponding to images **(A)**. The presented data are mean values derived from three biological replicates, with error bars indicating standard deviations. *P < 0.05 signifies a notable difference from the control group.

To verify the impact of ICS Ⅱ on oxidative stress, intracellular ROS generation was evaluated via ROS Brite™ 570 staining. As depicted in Figure 5, the relative fluorescent intensity of ROS Brite™ 570 exhibited a noteworthy reduction in the ICS Ⅱ-treated group compared to the control. These findings underscore the capacity of ICS Ⅱ to mitigate ROS production in HBV-positive cells.

## 4 Discussion

The current study unveils several crucial findings: (1) ICS Ⅱ, a naturally occurring active compound derived from Herbal EpimedⅡ, exhibits anti-HBV activity both *in vivo* and *in vitro*; (2) Our findings suggest that ICS II inhibits HBV replication by disrupting mitochondrial dynamics, potentially through a dual mechanism: (1) direct modulation of mitochondrial fission/fusion proteins (e.g., Fis1, Drp1), and (2) indirect effects on host cell redox balance. However, further experiments are needed to confirm whether these effects are mediated primarily through mitochondrial dynamics or other pathways.

Prior studies have found that the extract or polypill of EP has anti-HBV activities (Zhang et al., 2017; Tian et al., 2019; Liu et al., 2023). Accordingly, our inquiry sought to explore the anti-HBV capabilities of ICS Ⅱ, a principal active constituent of *Epimedium*. The influence of ICS Ⅱ on HBV replication was tested in an AAV-HBV mouse model. Our results indicated that ICS Ⅱ elicited no substantial effect on body weight, indicating that it has no significant toxic effect on mice. Notably, a significant reduction in serum HBsAg, HBeAg, and HBV DNA levels were observed in both ICS Ⅱ-treated groups, underscoring its potent anti-HBV effect. Correspondingly, our *in vitro* investigations using HBV-positive HepG2.2.15 cell lines reinforced these findings. Particularly striking was the superior anti-HBV effect of ICS Ⅱ observed in HepG2.2.15 cells compared to the positive control drug entecavir.

In this study, we confirmed the anti-HBV effect of ICS Ⅱ, but its mechanism is still unclear. Through transcriptome sequencing, we have identified 25 DEGs associated with mitochondria, among which Fis1 was one of the regulated gene. Fis1 is a key regulator of mitochondrial fission, and its upregulation by ICS II (Figure 4) aligns with the observed mitochondrial fragmentation in HBV-positive cells. This suggests that Fis1 may play a critical role in ICS II’s anti-HBV activity by promoting mitochondrial fission, thereby disrupting the mitochondrial network required for HBV replication.

Accumulating evidence underscores the pivotal role of mitochondrial dynamics in virus replication, offering a promising avenue for antiviral intervention (Kao et al., 2018; Proulx et al., 2021; Lee and Ou, 2022). Mitochondrial elongation induced by influenza virus, for instance, can be inhibited by Mito-C, a mitochondrial fragmentation activator, thereby curbing virus replication (Pila-Castellanos et al., 2021). Similarly, Mito-C attenuates dengue virus replication by countering virus-triggered mitochondrial fusion (Molino et al., 2020). A decade ago, it was demonstrated that HBV disrupt of mitochondrial dynamics towards fission and mitophagy curbs virus-induced apoptosis, potentially bolstering cell survival and viral persistence (Kim et al., 2013). In myocardial infarction, ICS II promoted mitochondrial fusion, and suppressed mitochondrial fission and oxidative stress (Li et al., 2023).

Consequently, we investigated the mitochondria labeled with MitoTracker Deep Red in HepG2.2.15 cells. ICS Ⅱ treatment resulted in a significant reduction in mitochondrial length, a phenomenon corroborated by TEM analysis. This implies an augmentation of mitochondrial fission and/or attenuation of fusion activities by ICS Ⅱ. Notably, a substantial increase in mitochondrial motility was observed in the ICS Ⅱ-treated group, suggesting that ICS Ⅱ may stimulate mitochondrial motility in HBV-positive cells.

The maintenance of a normal mitochondrial network and dynamic equilibrium relies upon a delicate interplay between fusion and fission processes. There are key proteins involved in regulation of these activities (Quintana-Cabrera and Scorrano, 2023). Notably, the promotion of mitochondrial fission is facilitated by Drp1 and Fis1, while the orchestration of mitochondrial fusion is driven by Optic Atrophy 1 (Opa1), Mitofusin 1 (Mfn1), and Mitofusin 2 (Mfn2) (Smirnova et al., 2001; Stojanovski et al., 2004; Landes et al., 2010; Sidarala et al., 2022). The upregulation of Fis1 and p-Drp1 S616, along with the downregulation of p-Drp1 S637, suggests that ICS II shifts the balance toward fission-dominant dynamics, which may impair mitochondrial function and energy metabolism, ultimately inhibiting HBV replication Given that HBV relies heavily on host cells for its replication, we conjecture that the disruption in mitochondrial dynamics, driven by ICS Ⅱ, reverberates onto cellular functions. Consequently, host cells might become incapable of providing a conducive environment for HBV replication, thereby impeding the progression of HBV replication.

The intricate role of reactive oxygen species (ROS) in modulating mitochondrial dynamics has been clarified, unveiling a connection between cellular redox equilibrium and the regulation of mitochondrial structure (Willems et al., 2015). Notably, HBV infection has been identified as a trigger for ROS accumulation (Yuan et al., 2016; Popa and Popa, 2022). Intracellular and mitochondrial ROS levels were higher in HepG2.2.15 cells than HepG2 cells and the lettuce extracts and luteolin-7-O-glucoside decreased ROS levels in HepG2.2.15 cells (Cui et al., 2017), and pu-erh tea extracts significantly reduces intracellular ROS levels in HepG2 2.2.15 cells (Pei et al., 2011). Knockdown of p53 reduced ROS levels and enhanced HBV replication in HepG2-NTCP cells, whereas p53 overexpression increased ROS and inhibited HBV replication in Hep3B-NTCP cells. The ROS scavenger N-acetyl-L-cysteine (NAC) reversed these effects (Jeong et al., 2024). The first-line chemotherapeutic drug 5-FU facilitates the HBV life cycle, and 5-FU treatment promoted the generation of ROS in HepG2.2.15 cells. ROS scavenger NAC reversed the stimulatory effects of 5-FU on ROS biosynthesis (Yang et al., 2024), which indicated that oxidative stress plays a critical role in HBV life cycle. The aforementioned studies indicate that various drugs or methods can exert anti-HBV effects by inhibiting ROS.

Published studies have demonstrated that ICS II exerts antioxidant effects across a spectrum of disease models. In PC12 cells, the levels of intracellular ROS increased significantly after exposed to H2O2 48 h, pre-treatment with ICS II (25-100 lM) suppressed H2O2-triggered ROS burst (Gao et al., 2017), and ICS II decreased ROS generation in 6-week-old Male C57BLKS/Leprdb mice (Li et al., 2022), and decreased ROS generation in myocardial infarction mice (Li et al., 2023). Remarkably, our present study revealed a conspicuous rise in ROS levels in HepG2.2.15 cells compared to HepG2 cells (The HepG2.2.15 cells was transfected by pDoLT-HBV in HepG2 cells, so we chose HepG2 as a HBV negative control.), yet this elevation was notably mitigated following ICS II treatment.

## 5 Conclusion

The current study marks a pioneering effort in unveiling the inhibitory impact of ICS Ⅱ on HBV replication, both *in vitro* and *in vivo*, accomplished through its adept modulation of mitochondrial dynamics and ROS production. This comprehensive exploration enhances our comprehension of the anti-HBV constituents within EP, while also illuminating a promising path for the continued advancement of ICS Ⅱ as a potential therapeutic intervention against HBV.

## Data Availability

The original contributions presented in the study are publicly available. This data can be found here: National Center for Biotechnology Information (NCBI) under the BioProject number PRJNA1241712.
