# Supplementary figures and images for: Icarisid Ⅱ modulates mitochondrial dynamics for anti-HBV activity
Source: Front Pharmacol. 2025 Apr 1;16:1544714. doi: 10.3389/fphar.2025.1544714 (PMC11996814; doi:10.3389/fphar.2025.1544714)

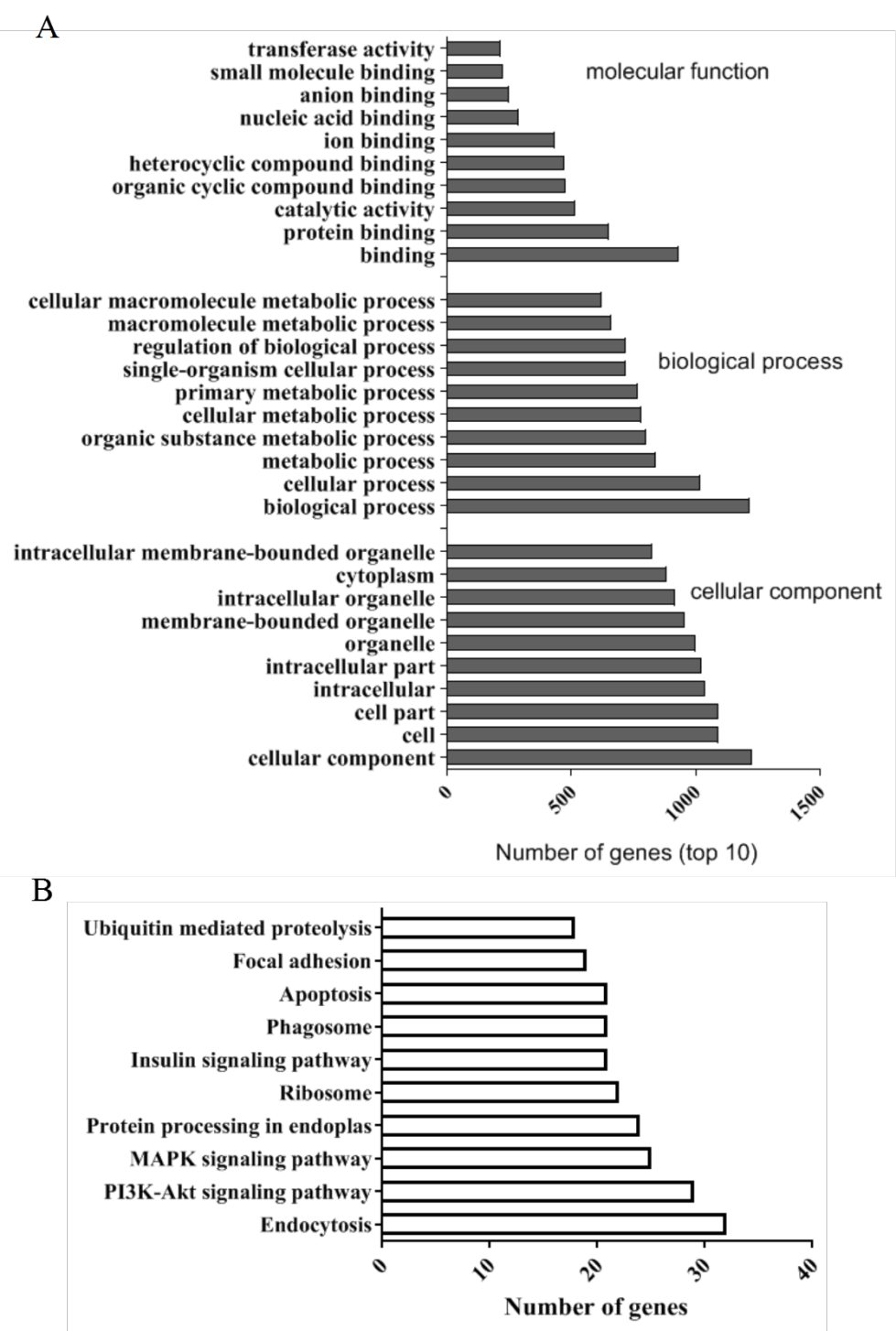

Supplement: Supplementary file 1 [file Image1.tif]
